# Supplementary material for: Long Noncoding RNA HCP5, a Hybrid HLA Class I Endogenous Retroviral Gene: Structure, Expression, and Disease Associations
Source: Cells. 2019 May 20;8(5):480. doi: 10.3390/cells8050480 (PMC6562477; doi:10.3390/cells8050480)
Supplement: Supplementary file 1 [file cells-08-00480-s001.zip › cells-509729 supplementary-5.20/_Table S1. Databases and repositories copy.pdf]

Table S1. Databases and Repositories

Comparative Toxicogenomics Database | CTD**:** CTD is a publicly available database that aims to advance understanding about how environmental exposures affect human health. It provides manually curated information about chemical–gene/protein interactions, chemical–disease and gene–disease relationships. These data are integrated with functional and pathway data to aid in development of hypotheses about the mechanisms underlying environmentally influenced diseases. http://ctdbase.org

Database of Genomic Variants: A curated catalogue of human genomic structural variation. http://dgv.tcag.ca/dgv/app/home

Database of HERV/LTR regulatory elements (dbHERV-Res): The database provides (i) general information on HERV/LTRs such as family classification, copy number, and insertion date judged by distribution of orthologous copies among mammalian genome; (ii) positions of HERV-TFBSs, HSREs, and HERV-DHSs in the consensus sequence of HERV/LTRs and in human reference genome; and (iii) results of Gene ontology (GO) enrichment analyses with the online computer program GREAT (http://bejerano.stanford.edu/great/public/html/) using sets of respective HSREs.

dbSNP short genetic variations: dbSNP contains human single nucleotide variations, microsatellites, and small-scale insertions and deletions along with publication, population frequency, molecular consequence, and genomic and RefSeq mapping information for both common variations and clinical mutations.
https://www.ncbi.nlm.nih.gov/projects/SNP, https://www.ncbi.nlm.nih.gov/snp/

ENCODE: The Encyclopedia of DNA Elements (ENCODE). The goal of ENCODE is to build a comprehensive parts list of functional elements in the human genome, including elements that act at the protein and RNA levels, and regulatory elements that control cells and circumstances in which a gene is active. The discovery and annotation of gene elements is accomplished primarily by sequencing a diverse range of RNA sources, comparative genomics, integrative bioinformatic methods, and human curation. Regulatory elements investigated through DNA hypersensitivity assays, assays of DNA methylation, and immunoprecipitation (IP) of proteins that interact with DNA and RNA, i.e., modified histones, transcription factors, chromatin regulators, and RNA-binding proteins, followed by sequencing. https://www.encodeproject.orgdbSUPER: dbSUPER is an integrated and interactive database of super-enhancers, which contains 82234 super-enhancers in 102 human and 25 mouse tissue/cell types (February 2009). https://www.encodeproject.org

Ensembl: Ensembl is a genome browser for vertebrate genomes that supports research in comparative genomics, evolution, sequence variation and transcriptional regulation. Ensembl annotate genes, computes multiple alignments, predicts regulatory function and collects disease data. http://asia.ensembl.org/index.html

E!Ensembl: Browser, automatically annotated genomes, comparative genomics, variation (dbSNP and DGVa) and regulatory data. https://asia.ensembl.org/info/about/index.html

EPDnew: Set of species-specific databases of experimentally validated promoters.
https://epd.epfl.ch//EPDnew_database.php

Gencode release 19. Annotation data set of gene features in the human genome using a combination of computational analysis, manual annotation, and experimental validation. https://www.gencodegenes.org/human/release_19.html

GEO datasets. This NCBI database stores curated gene expression DataSets, as well as original Series and Platform records in the Gene Expression Omnibus (GEO) repository. Enter search terms to locate experiments of interest. DataSet records contain additional resources including cluster tools and differential expression queries. https://www.ncbi.nlm.nih.gov/gds

GeneCard: A searchable, integrated, public database of human genes that provides concise genomic related information, on all known and predicted human genes. https://www.genecards.org

Harmonizome: Integrated Knowledge About Genes and Proteins: Connecting Big Data, collection of information about genes and proteins from 114 datasets provided by 66 online resources. http://amp.pharm.mssm.edu/Harmonizome/

Integrative DNA methylation database (iMethyl). Data of allele frequencies of SNVs, CpG methylation levels, and gene expression values of monocytes and CD4+ T cells from 102 individuals and other updated data. http://imethyl.iwate-megabank.org/index.html

NCBI: The National Center for Biotechnology Information is a public online resource that provides access to biomedical and genomic information.
https://www.ncbi.nlm.nih.gov

NCBI Gene: Gene integrated information from a wide range of species. A record may include nomenclature, Reference Sequences (RefSeqs), maps, pathways, variations, phenotypes, and links to genome-, phenotype-, and locus-specific resources worldwide.
https://www.ncbi.nlm.nih.gov/gene/

PathwayNet: Pathwaynet provides users the means to analyze their experimental gene hits in the context of predicted interaction networks incorporating tissue contextual information and refine regulatory interactions from ~52,000 primary experimental datasets (e.g. ChIP-Seq, mass spectrometry).
http://pathwaynet.princeton.edu/predictions/gene/?network=human-functional-relation&gene=16898

TCNG Cancer Network Galaxy Database. *Database of Cancer Gene Networks from Public Gene Expression Data*. http://tcng.hgc.jp

UCSF Browser: Browser, mapping and sequencing, genes and gene predictions, phenotypes and literature, comparative genomics, variation, regulatory data, expression, mRNA and EST, repeats. http://genome.ucsc.edu/cgi
