# Supplementary material for: Long Noncoding RNA HCP5, a Hybrid HLA Class I Endogenous Retroviral Gene: Structure, Expression, and Disease Associations
Source: Cells. 2019 May 20;8(5):480. doi: 10.3390/cells8050480 (PMC6562477; doi:10.3390/cells8050480)
Supplement: Supplementary file 1 [file cells-08-00480-s001.zip › cells-509729 supplementary-5.20/_Table S4. TranscriptionFactors copy.pdf]

Table S4. Internet databases with predicted HCP5 associated transcription factors (TFs).

TF datasets and (webpage)	No. TF
JASPAR Predicted Transcription Factor Targets (http://amp.pharm.mssm.edu/Harmonizome/dataset/JASPAR+Predicted+Transcription+Factor+Targets)	57
MotifMap Predicted Transcription Factor Targets http://amp.pharm.mssm.edu/Harmonizome/dataset/MotifMap+Predicted+Transcription+Factor+Targets	7
TRANSFAC Curated Transcription Factor Targets http://amp.pharm.mssm.edu/Harmonizome/dataset/TRANSFAC+Curated+Transcription+Factor+Targets	1
TRANSFAC Predicted Transcription Factor Targets http://amp.pharm.mssm.edu/Harmonizome/dataset/TRANSFAC+Predicted+Transcription+Factor+Targets	13
GeneHancer (GH) Regulatory Elements https://www.genecards.org/cgi-bin/carddisp.pl?gene=HCP5	11
